# The roles of cancer stem cell-derived secretory factors in shaping the immunosuppressive tumor microenvironment in hepatocellular carcinoma

**DOI:** 10.3389/fimmu.2024.1400112

**Published:** 2024-05-29

**Authors:** Gregory Kenneth Muliawan, Terence Kin-Wah Lee

**Affiliations:** ^1^ Department of Applied Biology and Chemical Technology, The Hong Kong Polytechnic University, Hong Kong, Hong Kong SAR, China; ^2^ State Key Laboratory of Chemical Biology and Drug Discovery, The Hong Kong Polytechnic University, Hong Kong, Hong Kong SAR, China

**Keywords:** cancer stem cell (CSC), hepatocellular carcinoma, immunosuppression, secretory factors, tumor microenvironment

## Abstract

Hepatocellular carcinoma (HCC) is one of the most prevalent malignancies worldwide and has a poor prognosis. Although immune checkpoint inhibitors have entered a new era of HCC treatment, their response rates are modest, which can be attributed to the immunosuppressive tumor microenvironment within HCC tumors. Accumulating evidence has shown that tumor growth is fueled by cancer stem cells (CSCs), which contribute to therapeutic resistance to the above treatments. Given that CSCs can regulate cellular and physical factors within the tumor niche by secreting various soluble factors in a paracrine manner, there have been increasing efforts toward understanding the roles of CSC-derived secretory factors in creating an immunosuppressive tumor microenvironment. In this review, we provide an update on how these secretory factors, including growth factors, cytokines, chemokines, and exosomes, contribute to the immunosuppressive TME, which leads to immune resistance. In addition, we present current therapeutic strategies targeting CSC-derived secretory factors and describe future perspectives. In summary, a better understanding of CSC biology in the TME provides a rational therapeutic basis for combination therapy with ICIs for effective HCC treatment.

## 1 Introduction to hepatocellular carcinoma

Cancer has long been a major public health concern, with consistently increasing incidence and mortality rates worldwide. According to GLOBOCAN estimation data for 2020, a total of 19 million new cancer cases and 10 million cancer-related deaths are predicted to occur globally (1). Primary liver cancer is the sixth most diagnosed cancer and the third leading cause of cancer-related deaths worldwide according to GLOBOCAN 2020 evaluations (1). Liver cancer has been established as one of the key contributors to the global cancer burden, and hepatocellular carcinoma (HCC) is one of the most commonly diagnosed histological subtypes of liver malignancy, accounting for approximately 80% of all liver cancer cases worldwide (2). Despite the significance of HCC cases in transitioning countries with low and medium resources, the tumorigenicity of HCC is significantly related to various risk factors and underlying liver disorders, including chronic hepatitis B virus (HBV) and hepatitis C virus (HCV) infections, nonalcoholic fatty liver disease (NAFLD), nonalcoholic steatohepatitis (NASH), compulsive alcohol abuse, smoking, aflatoxin exposure, metabolic syndrome, diabetes, and obesity. Approximately 75% of documented HCC cases are induced by HBV and HCV, rendering such infections crucial global risk factors for HCC (2). Furthermore, the contributions of both NAFLD and NASH to HCC progression are discernible in the evidence of common occurrences worldwide. Accumulating evidence has also demonstrated the causal linkage of risk factors, such as excessive alcohol and tobacco consumption, as well as underlying metabolic disorders, including obesity and diabetes, with an increased risk of HCC, including its precursors, NAFLD and NASH (3–6). HCC is a complex and heterogeneous malignancy known to have a poor prognosis, with the life prospects of individuals varying according to disease progression. More than 50% of HCC patients are diagnosed at an advanced stage of HCC development (7, 8). Individuals with advanced-stage HCC progression have an overall median survival rate of less than a year (8, 9), whereas those with early-stage HCC have a five-year median survival rate of approximately 70% with the corresponding treatment options (10).

## 2 Therapeutic options for hepatocellular carcinoma patients

The severity of HCC is acknowledged in both clinical and research settings, where various treatment modalities for this malignancy continue to be developed. The HCC staging systems, among which the Barcelona Clinical Liver Cancer (BCLC) is one of the renowned systems to be adopted, facilitate the selection of therapeutic options.

Liver transplantation is a highly effective treatment option for patients with early-stage HCC (BCLC 0/A) because it can remove tumor lesions and replace a damaged liver to prevent HCC recurrence (11–13). However, this treatment is recommended only for early-stage patients. Hepatic resection and ablation are other options that provide safe and effective outcomes for early-stage patients. Hepatic resection offers greater clinical benefits in terms of overall survival and tumor control but at a higher cost and with more adverse side effects than ablation techniques (14–16). Chemotherapeutic-based treatments, such as transarterial chemoembolization (TACE), are typically used for intermediate-stage HCC patients (BCLC B) with locally unresectable tumors.

The administration of tyrosine kinase inhibitors (TKIs) as a systemic therapy method is widely adopted to treat patients with HCC in both the intermediate and advanced stages (BCLC B/C). Sorafenib, an FDA-approved oral multikinase inhibitor, is capable of impeding HCC proliferation through the inhibition of numerous cell surface and downstream kinases that are involved in angiogenesis and tumor proliferation, including vascular endothelial growth factor receptor (VEGFR) 1–3, platelet-derived growth factor receptor (PDGFR) β, FMS-like tyrosine kinase-3 (FLT3), and c-KIT (17, 18). Additionally, the inhibition of Raf kinases, including B-Raf and C-Raf, which are implicated in the Ras/Raf/MEK/ERK signaling pathway, also inhibits HCC development. Similarly, another multikinase inhibitor, lenvatinib, was synthesized. This drug is a compelling inhibitor of fibroblast growth factor receptor (FGFR) 1–4, VEGFR 1–3, PDGFR-α, RET, and KIT. Unlike sorafenib, lenvatinib is capable of impeding the fibroblast growth factor (FGF) signaling pathway, which provides a basis for its antitumor activity (19–22).

Despite numerous therapeutic options, the clinical benefits for patients with HCC remain limited owing to adverse side effects, recurrence risk, and therapeutic resistance. TKIs such as sorafenib and lenvatinib are significant for advanced-stage HCC. However, primary and acquired resistance are major obstacles, and kinase inhibitors have become ineffective over time. Genetic diversity contributes to primary resistance, whereas acquired resistance can occur during treatment. A substantial body of research has identified crosstalk between multiple signaling pathways, including the PI3K/AKT, JAK/STAT, hypoxia-inducible, and epithelial–mesenchymal transition (EMT) pathways, as one of the primary sources of acquired resistance to sorafenib in HCC patients, along with other pathways (23–26). Similarly, some studies have shown that the signaling pathways that participate in acquired resistance to lenvatinib in HCC patients include the EGFR/PAK2/ERK5, PI3K/AKT, and MAPK/ERK pathway (27, 28). Ultimately, a wide range of additional molecular mechanisms contribute to the complex nature of therapeutic resistance in HCC patients.

Combination therapy that incorporates immunotherapies, including immune checkpoint inhibitors (ICIs) and chimeric antigen receptors (CARs), with conventional treatments, such as TKIs, locoregional and radical treatments, and neoadjuvant therapy, is ongoing. Immunotherapy, as a multifaceted treatment approach, has shown promising results in improving the treatment efficacy and clinical benefits for patients with liver cancer, especially HCC. However, investigating the molecular mechanisms leading to therapeutic resistance in patients with HCC is crucial for developing of effective treatments that provide substantial and consistent clinical benefits, especially for those in the advanced stages of the disease.

## 3 General concept of cancer stem cells

Tumor-initiating cells (TICs) or cancer stem cells (CSCs) are a subpopulation of malignant cells within the tumor bulk that can self-renew, differentiate, and form distinct tumor cell populations, leading to tumorigenesis, and are known to be involved in the development of therapeutic resistance in cancer patients. These cells are highly resistant to conventional chemotherapeutic therapies, rendering them resistant to apoptosis, able to survive and repopulate the tumor bulk with restricted proliferative potential, and metastasize to give rise to new tumors (29, 30). The intricate biological characteristics and mechanisms of these conspicuous cells further drive tumor aggressiveness and survival, which include but are not limited to metabolic alterations (31–33), redox stress tolerance (34, 35), prompt restoration of damaged DNA (36, 37), EMT signaling (38), hyperactivity and overexpression of ATP-binding cassette (ABC) transporters for drug efflux (39, 40), and the development of multidrug resistance (MDR) (40, 41).

The detection of CSCs is feasible due to the presence of intracellular and extracellular molecules that serve as markers, and these markers are also used for predictive, diagnostic, and therapeutic purposes. The adoption of combinations of markers may help to accurately isolate, enrich, and identify CSCs, as certain markers are shared by all types of malignant and nonmalignant cells (42). Transcription factors such as SOX2, OCT4, and NANOG, as well as surface markers such as CD24, CD34, CD44, CD90, CD123, and CD133, are commonly used to help distinguish CSC populations (43–45). For years, the presence of liver CSCs has been continuously investigated as one of the prominent mechanisms behind inefficient treatment outcomes and tumor relapse in liver cancer patients. Owing to extensive research performed throughout the years, numerous markers for isolating and characterizing CSCs in liver cancer have been discovered. Through evident xenotransplantation and fluorescence-activated cell sorting (FACS) experimental outputs, markers of CSCs in HCC can be classified into intracellular markers, such as cytokeratin 19 (CK19) and surface markers, including CD13, CD24, CD44, CD47, CD90, CD133, and epithelial cellular adhesion molecule (EpCAM) (46). These reported markers have been evaluated with regard to CSCs in HCC, which is known to be associated with poor patient survival and aggressive tumor development. However, the purpose of biomarkers is widely applied to characterize or label the CSC phenotype of various malignancies; for example, CD133 can be used as a CSC marker for liver, breast, lung, pancreatic, and prostate cancers (29). The combinatory use of several intracellular and extracellular markers has served as the foundation for the identification and isolation of CSC populations.

CK19 is a known intracellular marker for premature hepatoblasts, hepatic progenitor cells, and cholangiocytes, where it can serve as a biomarker for CSCs in HCC and is involved in the SMAD/transforming growth factor (TGF)-β signaling pathway (46, 47). Previous research has shown that CK19^+^ cells in HCC are associated with metastasis, drug resistance, poor prognosis, tumor differentiation, and tumor relapse (47–50). A research group reported that CK19 may be a predictive biomarker for tumor relapse following surgery and adjuvant therapy in patients with HBV-induced HCC (51). A similar study examined the association between the benefits of adjuvant TACE and CK19 expression and reported that CK19^+^ HCC patients with an elevated risk of tumor relapse may benefit from TACE in terms of recurrence-free survival (52). Moreover, it has been suggested that HCC patients with CK19^+^ cells could be treated with regorafenib, a dual-targeted VEGFR2-TIE2 TKI, for individualized therapeutic purposes (53).

The expression of CSC surface markers, including CD13, CD24, CD44, CD47, CD90, and CD133, is reported to be related to self-renewal, metastasis, proliferation, angiogenesis, poor prognosis, tumor recurrence, and drug resistance in HCC. These markers are also functional markers which regulate a number of signaling pathways (46, 54, 55). Recent studies have shown novel insights based on discoveries related to HCC cell surface markers, which are summarized in **Table 1**. Research groups have shown that CD13 expression is correlated with chemoresistance induction and sorafenib resistance through the p38/Hsp27/CREB/ATG7 and HDAC5/LSD1/NF-κB pathways, respectively (56, 57). Moreover, the suppression of CD13 expression by ubenimex, a CD13 inhibitor, may reverse chemoresistance in HCC, suggesting that combination therapy involving chemotherapeutic agents and ubenimex may be another treatment option to counter therapeutic resistance (58). Additionally, recent research demonstrated a novel method to impede CD24 expression through the use of RNAi technology that attenuated Salmonella and harnessed its ability to bind to oxaliplatin chemotherapeutic drugs (59). The application of this novel approach led to a decrease in stemness properties and an increase in T-cell infiltration, which increased antitumor immune responses. Moreover, a discovery revealed that CD24-related sorafenib resistance in patients with HCC is associated with the activation of autophagy, and the overexpression of CD24 leads to increased production of PP2A and promotes the downregulation of the mTOR/AKT pathway, which sustains autophagy (60). CD44 promotes HCC progression, metastasis, migration, and invasion through YAP (61), a downstream regulator of the Hippo pathway, the C-X-C motif chemokine receptor (CXCR) 4/Wnt/β-catenin pathway (62), and the CXCR4/AKT/ERK pathway (63). The obstruction of CD47 expression in a murine model has been shown to promote antitumor immunity through the CD103^+^ dendritic cell (DC)/natural killer (NK) cell axis (64). Moreover, a novel approach that incorporates a bispecific antibody that targets glypican-3 (GPC3) and CD47 has been shown to outperform combination therapy with anti-GPC3 and anti-CD47 in a xenograft HCC model (65). The involvement of CD90 in the biological functions of HCC remained unknown until a recent study revealed that CD90 participates in migrative, viability, and sphere-forming capabilities in HCC, indicating that CD90 may serve as a candidate for diagnostic and therapeutic approaches (66). Furthermore, a research group has targeted CD90 expression via miR-125a/b, which impedes tumor-associated macrophages (TAMs) mediated by CSCs in HCC (67). Recently, several research groups have made innovations in therapeutic strategies targeting CD133 for tumor development and preservation. Novel applications of therapeutic compounds, including chromenopyrimidinone (CPO), oxytetracycline, a CD133-specific aptamer conjugated with doxorubicin, and actinomycin D (ActD), were found to suppress the expression of CD133, which leads to a positive regulation of the stemness of HCC cells (68–71).

**Table 1:** Summary of CSC surface markers and their roles in HCC.

Marker Name	Research Output	Study Design	Reference
CD13	CD13 enhances HCC chemoresistance through p38/Hsp27/CREB/ATG7 pathway	*In vitro* HCC cell culture; *In vivo* mice model	(56)
	CD13 promotes sorafenib resistance through HDAC5/LSD1/NF-kB pathway	*In vitro* HCC cell culture; *In vivo* mice model	(57)
	CD13 inhibitor (ubenimex) in combination with chemotherapeutic agents combats HCC chemoresistance	*In vitro* HCC cell culture; *In vivo* mice model; HCC patient sample	(58)
CD24	CD24 inhibitor (siRNA-CD24) based on attenuated salmonella enhances oxaliplatin effectiveness on HCC	*In vitro* HCC cell culture; *In vivo* mice model	(59)
	CD24 regulates sorafenib resistance through PP2A production and deactivates mTOR/AKT pathway	*In vitro* HCC cell culture; *In vivo* mice model; HCC patient sample	(60)
CD44	CD44 promotes hepatocarcinogenesis through Hippo/YAP signaling	*In vitro* HCC cell culture	(61)
	CD44 regulates HCC stemness through CXCR4/Wnt/β-catenin axis	*In vitro* HCC cell culture	(62)
	CD44 modulates HCC stemness through AKT/ERK signaling CXCR4 axis	*In vitro* HCC cell culture; *In vivo* mice model; HCC patient sample	(63)
CD47	CD47 inhibition promotes anti-tumor immune response through CD103^+^ DC-derived IL-12 and CXCL9 and NK activation	*In vivo* mice model	(64)
	CD47/GPC3 bispecific antibody promotes anti-tumor efficacy	*In vitro* HCC cell culture; *In vivo* mice model	(65)
CD90	CD90 regulates cancer stemness properties of HCC cells	*In vitro* HCC cell culture; HCC patient sample	(66)
	CD90 impediment by miR-125a/b decreases stemness properties of HCC cells	*In vitro* HCC cell culture	(67)
CD133	CD133 suppression by chromenopyrimidinone (CPO) reduces stemness properties of HCC cells	*In vitro* HCC cell culture; *In vivo* mice model; Molecular docking	(68)
	CD133 inhibition by oxytetracycline through protein destabilization suppresses HCC stemness	*In vitro* HCC cell culture; *In vivo* mice model	(69)
	CD133-specific aptamer conjugated with doxorubicin inhibits hepatocarcinogenesis	*In vitro* HCC cell culture; *In vivo* mice model	(70)
	CD133 repression by actinomycin D (ActD) reduces stemness and malignant properties of HCC cells	*In vitro* HCC cell culture	(71)

Aside from the expression of CSC markers in HCC, several intrinsic regulatory molecules have also been reported to play a role in tumor initiation and self-renewal capabilities in HCC (46). These molecules include long noncoding RNAs (lncRNAs), microRNAs (miRNAs), epigenetic regulators, kinases and phosphatases, metabolic regulators, transcription factors, and secretory molecules. Generally, these elements congregate into common renowned signaling pathways, including the MAPK, JAK/STAT, IL-6/STAT3, TGF-β, NF-κB, JNK, Sonic Hedgehog (SHH), Wnt/β-catenin, and Notch pathways. Ultimately, these signaling pathways are associated with the HCC tumor microenvironment (TME), which regulates CSCs, along with other elements, including cancer-associated fibroblasts (CAFs) and TAMs (54, 55). As reciprocal interactions between CSCs and their niches allow the formation of an immunosuppressive microenvironment, the preceding intrinsic regulators of CSC properties may also contribute. Several lncRNAs and miRNAs can exert immunosuppressive influence to facilitate HCC progression, such as lncMALAT1 (72), lncHULC (73), lncHOTAIR (74), lncLINC01132 (75), lncPVT1 (76), lncLINC00662 (77), lncβ-Catm (78), miR-23a-3p (79), and miR-146a-5p (80). Dysregulated expression of epigenetic regulators, including DNMT3α (81), KDM1A (82, 83), YTHDF2 (84), and RALYL (85), as well as kinases and phosphatases such as IRAK1 (86) and SHP2 (87), may also contribute to the formation of an immunosuppressive TME in HCC. As an intricate malignancy, HCC is characterized by metabolic reprogramming, in which glycolytic and lipid metabolism aberrations can contribute to an immunosuppressive TME. The implicated metabolic regulators include XOR (88), SCD1 (89), and ACLY (90). Finally, transcription factors involved in regulating cancer stemness in HCC, such as SALL4 (80) and FOXM1 (91), are known to exert immunosuppressive effects on the TME of HCC.

## 4 Concept of secretome and secretory factors within the TME

The secretome can be defined as a biological factor, either soluble or insoluble, that is secreted or released into the extracellular space by cells, tissues, organs, and organisms. The secretome consists of growth and coagulation factors, chemokines, cytokines, hormones, glycoproteins, miRNAs, and enzymes (92, 93). These molecular or biological factors are secreted through mechanisms involving constitutive and regulated secretory organelles at any given time. In healthy cells, a subtly regulated secretome is crucial for the maintenance of physiological tissue homeostasis and corresponds to a large proportion of synthesized proteins in secretory tissues, along with their associated receptors, which operate as the key mechanism in cell-to-tissue communication (92, 94). The dynamic composition of the secretome is usually based on the cell type and stimulus from the microenvironment, where it possesses therapeutic benefits that are valuable for repairing impaired tissue (94, 95). Numerous biologically active molecules within the secretome exert therapeutic benefits, ranging from anti-inflammatory and immunomodulation (e.g., interleukin (IL)-6, IL-8, hepatocyte growth factor (HGF), prostaglandin E2 (PGE2), TGF-β), antifibrotic (e.g., VEGF, HGF, TIMP-1, TIMP-4, FGF-2), antiapoptotic (VEGF, HGF, TGF-β, stanniocalcin (STC)-1, FGF) and proangiogenic effects (e.g., VEGF, HGF, FGF-2, IL-6, IL-8, TGF-β, monocyte chemoattractant protein (MCP)-1) (95). Similar to healthy tissues, the tumor secretome is composed of an abundant number of elements, including growth factors (e.g., TGF-β, VEGF, HGF), immunosuppressive cytokines (e.g., IL-4, IL-10, CCL22, CCL28, CXCL12, CCL5), and other components, such as enzymes [e.g., indoleamine 2,3-dioxygenase (IDO), cyclooxygenase-2 (COX-2)], glycoproteins [e.g., galectin (GAL)1, GAL3, alpha-fetoprotein (AFP)] and EVs (e.g., tumor-derived EVs carrying PGE2, TGF-β, adenosine, IDO, GALs) (92–94). These factors are important intermediaries of cell-to-cell interactions, where augmented patterns of secretion are associated with the regulation of cancer hallmarks such as metastasis, drug resistance, tumor development, and angiogenesis. There are various pathways and mechanisms that are implicated in maintaining the tumor secretome, including genetic mutations (e.g., c-Myc, p53, PTEN), the regulation of miRNAs, and the influence of the cellular microenvironment (e.g., hypoxic conditions) (96).

**Figure 1:**
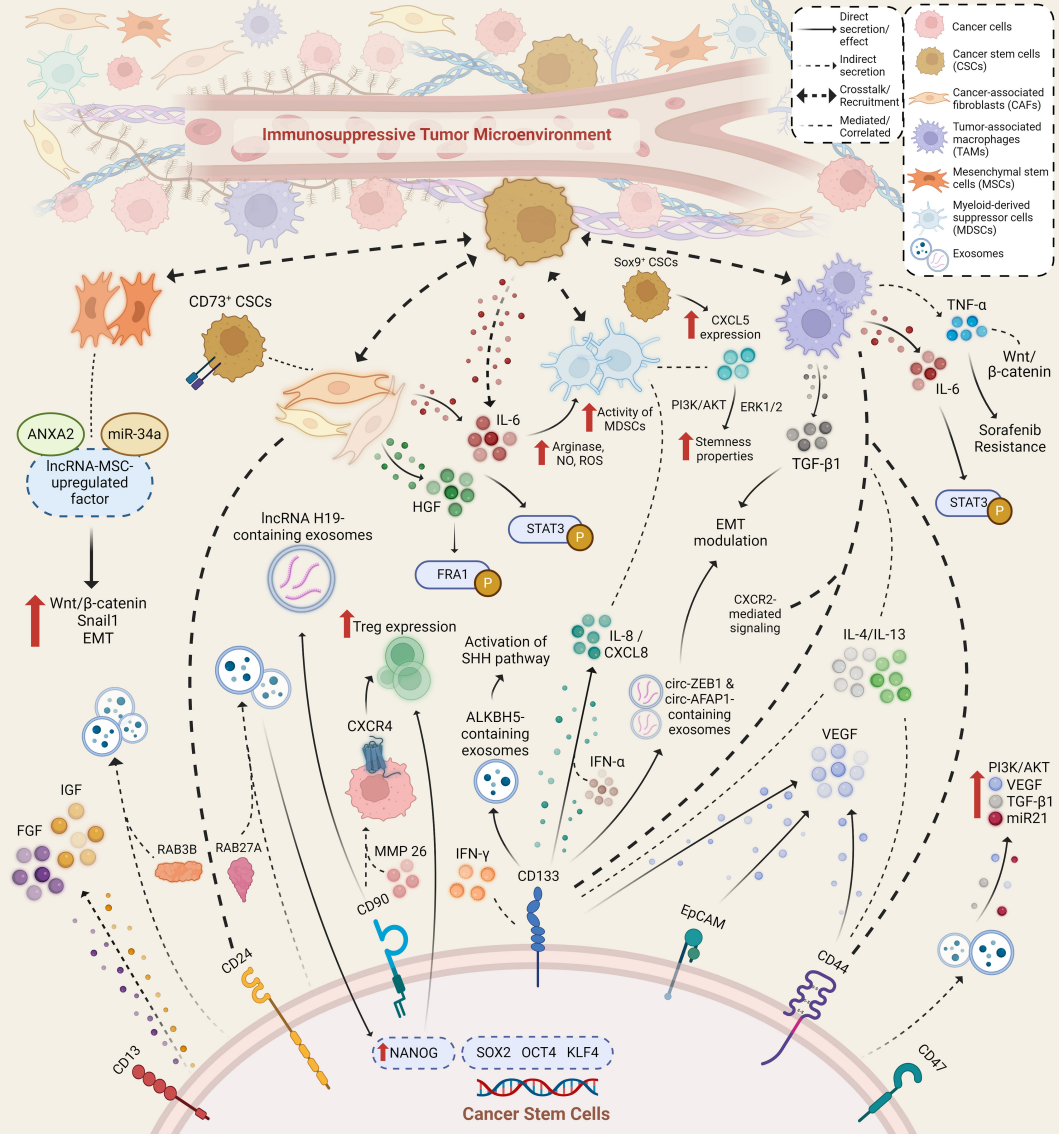
Immunosuppressive secretory factors that are secreted and/or mediated by cancer stem cells and other stromal and immune cells within the TME of HCC. Various cell types secrete and mediate the secretion of various secretory factors within the TME through direct and indirect method, where possible crosstalk and recruitment amongst secretory factors and cell types occur. Aside from exerting immunosuppressive capabilities, secretory factors also lead to an increase in stemness properties and hepatocarcinogenesis through activated signaling pathways and altered expression of various factors.

Normal adult stem cell tissues are primarily located within or adjacent to an *in vivo* microenvironment known as a “niche,” which comprises an assortment of cell types ranging from immune, endothelial, perivascular, and fibroblastic cells, along with the presence of extracellular matrix (ECM) components, growth factors, and cytokines. The interplay of components that exist in the niche maintains the stemness of stem cells, and the niche itself also facilitates the reception of extrinsic signals to induce stem cell performance (92). Similarly, within the TME, CSCs dwell and adapt to structurally distinct niches with components that favor CSC phenotypic properties of self-renewal and differentiation, as well as their metastatic potential and therapeutic resistance. CSCs may contribute to the secretion of various factors, either directly or by other means of crosstalk, recruitment, and stimulation of various cellular components, such as CAFs, MSCs, and immune cells. Ultimately, these secretory factors are components of the CSC niche. The reciprocal interactions between CSCs and such components contribute to the regulation of cancer hallmarks and alter the immune regulatory system, leading to the formation of an immunosuppressive TME. Previous studies have revealed that in HCC, the interplay of TME components with cancerous cells can induce liver fibrosis and promote HCC progression. Therefore, understanding how CSC-related secretory factors influence and help shape an immunosuppressive TME in HCC may ultimately benefit the development of effective and safe treatment options for HCC. Several studies have provided evidence related to immunosuppressive factors, ranging from cytokines, growth factors, and exosomes, which are secreted and/or mediated by CSCs and other components within the TME (**Figure 1**).

## 5 Effects of CSC-associated cytokines in shaping an immunosuppressive TME

Cytokines are a superfamily of proteins that are mainly produced by leukocytes during phases of naturally acquired immunity and have the ability to modulate inflammatory responses and the immune system. Cytokines are also involved in the positive and negative regulation of cellular activities, including growth, apoptosis, differentiation, and migration. These small glycoproteins are also secreted from different sources and may not regulate nonimmune-related events in various tissues. In the context of cancer, the function and mode of action of cytokines have been extensively studied in an attempt to exploit their antitumorigenic abilities for immunotherapeutic advancements. Cytokines normally operate by alerting the immune system to tissue injury and infection, although constant stimulation of cytokine production and secretion by immune cells may result in long-lasting inflammatory conditions and tumor development (97). Malignant cells are capable of secreting cytokines during cancer development to establish an immunosuppressive TME, where these cytokines act synergistically with other cell types to inhibit antitumorigenic responses and stimulate protumorigenic signals. Cytokines can be categorized as proinflammatory, anti-inflammatory, or immunosuppressive cytokines, the latter of which can impede the synthesis and control-related cellular activities of proinflammatory cytokines. However, owing to the pleiotropic actions of several cytokines, which promote an array of functional reactions from various cell subtypes, they are not categorized as either pro- or anti-inflammatory cytokines (98). The impact of cytokines on the development of cancer is influenced by several factors, including the concentration of cytokines, the tumor microenvironment (TME), the expression of receptors, and the balance between pro- and antitumorigenic cytokines. Different categories of cytokines, such as interleukins, chemokines, interferons (IFNs), and tumor necrosis factor (TNF), are considered in this discussion.

### 5.1 Interleukins

Interleukins, a subtype of cytokines, play an essential role in the activation and differentiation of immune cells and regulate growth and activation during immune responses and inflammation. In cancer, ILs play a role in fostering a conducive TME that promotes tumor development, self-renewal, survival, and immunosurveillance modulation. HCC is a malignancy closely associated with inflammation; however, the systemic interplay between ILs and HCC is still not well defined. Moreover, ILs play an essential role in facilitating crosstalk between CSCs and TME components. Certain ILs, such as IL-1, IL-6, and IL-8, are known to regulate the growth of CSC populations in various malignancies through bidirectional communication (99, 100).

The secretion of IL-6 by HCC cells with a chemoresistant phenotype, allegedly CSCs, has been reported to increase the development and activity of immunosuppressive MDSCs, in which high levels of immunosuppressive factors, namely, arginase, nitric oxide (NO), and reactive oxygen species (ROS), were observed (101). This finding highlights the pleiotropic nature of IL-6, which may contribute to the formation of an immunosuppressive TME. Furthermore, it was reported that CD133^+^ CSCs of HCC cells favorably secrete IL-8, which affects surrounding cells within the CSC niche, resulting in angiogenic effects (102). It was discovered that IL-8 is likely secreted by liver CSCs, as a report demonstrated a positive correlation between the CSC enrichment ratio and NANOG expression with IL-8 secretion (103). The inhibition of IL-8 repressed the properties of HCC CSCs and greatly improved cell sensitivity to sorafenib treatment (104). Although there is a lack of convincing evidence that IL-8 acts as an immunosuppressive cytokine, this chemoattractant may be capable of conditioning the TME to become immunosuppressive through the recruitment of myeloid-derived suppressor cells (MDSCs) to the TME. Studies have also revealed that IL-4 and IL-13 may contribute to the creation of an immunosuppressive TME, as they recruit and activate TAMs and MDSCs. A study, which used cell line models that originated from the AKT1/NRAS-induced HCC mouse model, revealed that IL-4/IL-13 signaling plays a crucial role in cancer development and that these cells exhibit strong CSC and CD44^+^ and CD133^+^ signatures (104). There is a lack of evidence that CSCs in HCC are involved in the secretion of both IL-4 and IL-13, although some studies have shown that CSCs in other malignancies can secrete these immunosuppressive cytokines (105).

### 5.2 Chemokines

Chemokines are secreted proteins that play essential roles in organ development and immune responses by binding to their corresponding receptors. Chemokines are involved in the coordination and recruitment of immune cells from and toward tissues, as well as in modulating interactions among immune cells. In the TME, chemokines regulate the growth, invasiveness, and stemness of tumor cells, and, simultaneously with their production by tumor cells, chemokines attract leukocytes, modulate neurogenesis and fibrogenesis, and induce the formation of blood vessels (106). Nevertheless, how chemokines operate within the TME to modulate several elements of immune cell initiation and phenotypic distinction remains unclear. The expression of numerous types of chemokines and their receptors has been characterized and studied in HCC cells (107).

From a previously described study, elevated expressions of chemokines, including CXCL2, CXCL3, CXCL15, CXCL17, CXCR2, and CXCR4, within the TME of established cell lines models that derived from AKT1/NRAS-induced HCC mouse model has also been reported (104). Studies have shown that the CXCR4/CXCL12 axis has an essential biological function and contributes to tumor metastasis and angiogenesis in various malignancies, including HCC (108). Because CSCs can promote metastasis and tumor recurrence, the CXCR4/CXCL12 axis may indirectly influence the properties of CSCs. After a series of subcutaneous implantations involving NOD/SCID mice, it was revealed that circulating HCC cells in the presence of CXCR4 along with CD90 and matrix metalloproteinase (MMP) 26 exhibit the CSC properties of tumor formation and metastatic potential (109, 110). The immunosuppressive effect of CXCR4 on the TME is known to be due to its high expression in a subset of immunosuppressive regulatory T cells (Tregs). It has also been shown that the inhibition of CXCR4 results in a reversal of Treg suppression, hence enhancing antitumor immune responses (111). CXCL5 expression is induced by Sox9^+^ HCC CSCs, which triggers HCC migration, invasion, and proliferation through the PI3K-AKT and ERK1/2 signaling pathways (112). CXCL5, a well-known chemokine, is assumed to contribute to the establishment of an immunosuppressive TME and tumor stimulation through the recruitment and activation of MDSCs (113). A recent study revealed that CD133^+^ HCC CSCs secrete high levels of CXCL8, which is preferentially induced by IFN-α and leads to the induction of immunosuppressive effects (114).

### 5.3 Interferons

IFNs are a family of pleiotropic cytokines that possess antitumor, antiviral, and immunoregulatory effects and are generally categorized into three groups, namely, alpha, beta, and gamma, corresponding to their cellular source. Type 1 IFNs include IFN-α and IFN-β, which are commonly secreted by virus-infected cells, and type 2 IFNs include IFN-γ, which mainly originates from immune cells, such as macrophages, NK cells, and T cells. In the context of cancer, IFN-γ has a dual role as an antitumorigenic factor and protumorigenic factor, where it can promote immune evasion and the establishment of an immunosuppressive TME (115). In HCC, a positive association between CD133^+^ CSCs and IFN-γ was established, where CD133^+^ CSCs were able to selectively resist IFN-γ-mediated apoptosis and autophagy (116). In a recent study, IFN-γ was also found to increase the CSC ratio and properties in HCC cells, as well as increase CSC resistance to sorafenib treatment through ferroptosis inhibition (117). Moreover, sensitization to sorafenib-mediated ferroptosis by PD-L1 knockdown led to the inhibition of IFN-γ-related CSCs in HCC (117). These findings demonstrate the ability of IFN-γ to promote an immunosuppressive TME in HCC.

### 5.4 Tumor necrosis factors

Tumor necrosis factors are versatile cytokines that exist in two forms, TNF-alpha (TNF-α) and TNF-beta (TNF-β), which are involved in various cellular processes, including immune modulation, inflammatory responses, and cell death. TNF-α is a widely studied cytokine due to its crucial role in inflammation-associated tumorigenesis, during which its release primarily involves a variety of immune cells, such as macrophages, dendritic cells (DCs), and T cells. Like other cytokines, TNF-α exhibits pleiotropic effects, which can have both antitumor and protumorigenic effects, as observed in HCC. TNF-α is implicated in hepatocarcinogenesis and HCC tumor relapse by activating hepatic progenitor cells (118) but also contributes to sorafenib resistance through the induction of EMT (119). To date, there is no research indicating the direct secretion of TNF-α by CSCs in the HCC TME; however, the crosstalk between CSCs and TAMs may cause TNF-α release.

## 6 CSC-mediated immunosuppressive growth factors

As secreted biologically active elements, growth factors act on specific cell receptors that successively transmit their growth signals to other intracellular molecules, eventually resulting in modified gene expression. The tumorigenic role of growth factors in various cancers, including HCC, continues to be explored, and these factors are known to stimulate major signaling pathways involved in promoting tumor development, angiogenesis, and therapeutic resistance. Both TGF-β and vascular endothelial growth factor (VEGF) are major growth factors with immune-suppression capabilities that can regulate the TME in HCC. Robust evidence has demonstrated the hepatocarcinogenic role of TGF-β, which is secreted by HCC cells in general; however, there is no direct evidence indicating that HCC CSCs are responsible for TGF-β release (120). In contrast, VEGF is likely secreted by CSC-like HCC cells, which exhibit high expression of the stemness markers CD44, CD133, and EpCAM following sublethal heat treatment to mimic incomplete radiofrequency ablation treatment (121). In the same study, VEGFR-1 promoted the stemness of treated HCC cells, which exhibited enhanced metastatic and migratory potential (121). Moreover, activation of insulin-like growth factor (IGF) and FGF signaling cascades was correlated with the development of CSC-enriched sorafenib-resistant HCC cells (122). It has been demonstrated that members of the IGFs and FGFs can potentially possess immunosuppressive capabilities, despite having contextual proinflammatory effects (123–126), which in this case may contribute to the formation of an immunosuppressive TME in HCC.

## 7 Immunosuppressive role of CSC-derived exosomes

A subset of extracellular vesicles (EVs), known as exosomes, are nanoscale particles that are actively released by various cell types, such as epithelial cells, immune cells, neuronal cells, and tumor cells. Biomolecular components of exosomes, namely, lipids, proteins, nucleic acids, and metabolites, are transferred between cells either locally or in distant microenvironments. During cancer development, this process mediates cell-to-cell communication, which is correlated with cancer hallmarks (127). Correspondingly, exosomes that originate from CSCs are secreted by malignant cells and act as mediators to permit communication with the TME. The biological features of these exosomes allow them to participate in cancer progression, proliferation, angiogenesis, metastasis, chemoresistance, and recurrence, suggesting that cancer-derived exosomes are potential biomarkers for various malignancies (127, 128). Moreover, these exosomes can modulate and alter the establishment of an immunosuppressive TME, facilitating the exchange of information from CSCs and promoting immune evasion mechanisms.

As a member of the small GTPase superfamily and Rab family, RAB27A is known to regulate the discharge of exosomes from cells. It was demonstrated that HCC cells with high metastatic capability can release exosomes that are reliant on RAB27A, which ultimately trigger EMT in low metastatic cells (129). RAB27A-mediated exosome secretion from liver CSCs cells leads to an increase in NANOG expression and resistance to regorafenib (129). An elevated signature of NANOG may increase the expansion of Tregs, which contributes to the formation of an immunosuppressive TME (130) and facilitates immune escape, particularly from NK cells (131). In another study, exosomes derived from CD133^+^ CSCs that contained ALKBH5 were found to be related to SOX4 and to activate the SHH signaling pathway, possibly leading to an immunosuppressive TME in HCC (132). An extensive *in vivo* study involving an HCC albino rat model revealed that CSC-derived exosomes enhanced tumor metastasis and growth through elevated expression of immunosuppressive factors, including TGF-β1, VEGF, miR-21, and the PI3K pathway (133). RAB3B-induced exosome secretion by HCC cells exhibiting stemness properties may indirectly contribute to the formation of an immunosuppressive TME, as indicated by a positive correlation with the expression of apolipoprotein E, which bears immune suppression capabilities. Further elucidation is required to validate such association (134).

Moreover, researchers have also revealed the role of exosomal biomolecules that may drive the secretion of CSC-derived exosomes within the TME. CD90^+^ liver CSCs secrete exosomes that contain lncRNA H19, which regulates endothelial cells and induces angiogenic properties that influence the TME (135). Although unrelated to HCC, lncRNA H19 could have an immunosuppressive effect on the TME of gastric cancer cells (136). CD133^+^ liver CSCs were found to secrete exosomes containing circ-ZEB1 and circ-AFAP1, which may be involved in the crosstalk between non-CSCs and CSCs of HCC and can modulate EMT activity (137). In HCC, EMT is known to participate in the establishment of an immunosuppressive TME, elevated tumor heterogeneity, drug resistance, and metastasis.

Several studies have reported that the direct secretion of various secretory factors from liver CSCs facilitates the formation of an immunosuppressive TME. However, some findings were correlated with the influence of TME components on the stemness properties of HCC cells, which has also been described. The complexity of HCC and the dynamic nature of the secretion of factors by CSCs pose additional challenges for further elucidation of the secretion of factors by CSCs within the HCC TME. For instance, IL-10 is a well-known immunosuppressive cytokine that is commonly produced by various immune cells, but direct evidence demonstrating the ability of CSCs in HCC to secrete IL-10 has not been found. Instead, immunosuppressive factors may be released by CSC-associated TME components.

## 8 Crosstalk and recruitment of TME components by CSC-derived secretory factors

Accumulating evidence suggests that, in addition to the secretion of factors by CSCs into their surroundings, CSCs are also involved in the recruitment, activation, and crosstalk of various cellular components within the CSC niche to modulate the immunosuppressive TME. Such components may include but are not limited to CAFs, undifferentiated mesenchymal stem cells (MSCs), and immune cells, such as TAMs and MDSCs. These cellular components serve as key players in the TME to preserve tumor growth; maintain CSCs, angiogenesis, migration, and metastasis; regulate inflammatory processes; and produce ECM components (138).

### 8.1 Cancer-associated fibroblasts

The development of CAFs in the TME originates from fibroblast modifications via crosstalk with malignant cells and other stromal cells, which ultimately promotes regional tumor invasion, progression, and dedifferentiation through the influence of elements such as chemokines, growth factors, and ECM-modifying metalloproteases. These highly inflamed cells play a substantial role in HCC progression, as the majority of patients with HCC have cirrhosis and a large number of stimulated fibroblasts. The crosstalk between CD24^+^ HCC CSCs and CAFs results in the paracrine secretion of HGF/c-Met and IL-6/IL-6R, which results in an enhancement of stemness properties through the phosphorylation of STAT3 (139). In this study, the pleiotropic nature of HGF and IL-6 was shown to be involved in the activation of STAT3 signaling, which may facilitate the formation of an immunosuppressive HCC TME. Similarly, another study demonstrated that the crosstalk of CAFs with CSCs led to the significant secretion of CAF-derived HGF and FRA1 activation (140). The secretion of HGF, which induces the activation of FRA1, might have an indirect facilitative influence on creating an immunosuppressive TME in HCC. Moreover, another study revealed the possible synergistic effect of CAFs with CD73^+^ CSCs, which promotes HCC tumorigenesis and chemoresistance through paracrine secretion of HGF from CAFs (141).

### 8.2 Mesenchymal stem cells

As one of the precursors of CAFs, undifferentiated MSCs are associated with the progression of malignancies and metastatic events, as well as an increase in the abundance and tumorigenic potential of CSCs. In HCC, accumulating research has revealed the controversial role of MSCs in the dual characteristics of tumor induction and suppression via positive regulation of the immune system. Recently, MSCs of diverse origins have been shown to be correlated with HCC progression and metastasis. Crosstalk between CSCs and HCC-associated MSCs may involve a novel lncRNA-MSC-upregulated factor, which was found to be highly expressed and associated with HCC tumorigenicity (142). Implicitly, lncRNA-MSC-upregulated factors, linked with ANXA2 and miR-34a, resulted in the activation and upregulation of immunosuppressive signaling pathways, namely, Wnt/β-catenin, Snail1, and EMT, which may facilitate the formation of an immunosuppressive TME in HCC (142).

### 8.3 Tumor-associated macrophages

In general, macrophages can polarize into two phenotypes, proinflammatory M1 and anti-inflammatory M2 macrophages, in which TAMs express M2 properties, leading to immunosuppression and protumorigenic capabilities. TAMs are immune cells within the tumor stroma that induce cancer progression and support the CSC niche. These well-defined cells are recruited by CSCs within the HCC tumor bulk via chemokine secretion. The crosstalk mechanism between TAMs and CSCs was shown to be related to the immunosuppressive EMT process induced by TAM-derived TGF-β1 (143). Moreover, M2 TAMs were found to be related to CD44^+^/CD133^+^ sorafenib-resistant HCC cells, which correlated with potential immunosuppressive CXCR2 signaling (144). Crosstalk between CD133-expressing HCC cells and TAMs could lead to the promotion of stemness properties, EMT activity, and the secretion of immunosuppressive TNFα, which are correlated with the Wnt/β-catenin pathway (145). In addition, two studies revealed that the interaction of TAMs with HCC CSCs and various markers led to the secretion of IL-6 by TAMs and subsequently activated STAT3 signaling (101, 146).

### 8.4 Myeloid-derived suppressor cells

In addition to TAMs, which are crucial immunomodulators of the TME, MDSCs play a prominent role in inhibiting T-cell infiltration into tumor sites. These premature marrow-derived cell populations contribute to HCC progression through either modulation of T-cell responses or independent action. MDSCs exert their immunosuppressive function by suppressing T-cell immune responses through the production of NO, ROS, arginase 1, IL-10, and TGF-β (147–149). The stimulation of immune suppressor cells, such as regulatory T-cells, through differentiation and expansion can further impede T-cell activity (148, 149). In terms of HCC, MDSCs can impede the cytotoxic function of NK cells against HCC cells and diminish the production of IFN-γ through membrane-bound TGF-β1 (148). MDSCs are strongly associated with therapeutic resistance to ICIs. In terms of HCC, a study revealed that MDSCs might be implicated in anti-PD-L1 resistance, where HCC patient-derived T-cells were found to be exhausted and that PD-L1+ MDSCs are involved in the suppression of T-cell function (150). An additional study revealed that MDSC-induced CCRK/EZH2/NF-κB/IL-6 signaling in HCC facilitates dampened antitumor T-cell activity and leads to immune evasion (151). As CCRK depletion resulted in a decrease in the immunosuppressive effect of MDSCs and led to an increase in anti-PD-L1 efficacy, this may suggest the influence of MDSCs on anti-PD-L1 resistance in HCC (151). To date, there is no related evidence indicating that the recruitment and crosstalk between MDSCs and CSCs in HCC leads to the secretion of immunosuppressive secretory factors. However, several studies have mentioned the possibility that factors within the TME that participate in MDSC recruitment may drive tumorigenesis. These factors include IL-1α (152), HIF-mediated IL-1β (153), HIF-mediated CCL26 (154), CCL2 (155), CXCL1 (155), CD36^+^ CAFs (156), CCL5 (157), HMGB1 (158), and GM-CSF (159).

## 9 Therapeutic implications of targeting secretory factors within the immunosuppressive TME of HCC

The complex interplay between tumor cells, CSCs, cellular elements, and immune cells within the TME presents a challenge for therapeutic intervention. CSCs exert intricate therapeutic resistance mechanisms, which leads to difficulties in overcoming the immunosuppressive TME of HCC. The development of innovative approaches to counter the immunosuppressive TME, which mostly involves the inhibition of allegedly implicated secretory factors, is imperative. While most of these inhibitors or molecules have already been assessed by researchers in the preclinical stage, some are undergoing clinical trials (**Table 2**).

**Table 2:** Therapeutic strategies that target immunosuppressive secretory factors within the TME of HCC.

Molecule/Agent/Inhibitor	Therapeutic outcomes / predicted effects	Stage (Pre-Clinical, Clinical, Approved)	Reference
Anti-Gr-1 antibody	Targets MDSCs and inhibits the levels of IL-6 induced immunosuppressive elements (Arginase, NO, ROS)	Approved	(101)
BMS-986253 + Nivolumab	An improved radiographic objective response rate through IL-8 blockade over Nivolumab monotherapy in patients with advanced HCC	Clinical Trial Phase II (NCT04050462)	(160)
BMS-813160 (Dual CCR2/CCR5 antagonist) + BMS-986253 + Nivolumab	Significant immune response against HCC and an improved long term survival rate in HCC patients through CCR2/CCR5 dual blockade	Clinical Trial Phase II (NCT04123379)	(161)
HuMAX-IL8	Blockade of IL-8 reduces mesenchymal phenotypes in tumor cells that may diminish treatment resistant actions	Clinical Trial Phase I (NCT02536469)	(162)
CHS-388	Safe, well-tolerable, and effective mono-therapeutic approach that targets IL-27 in HCC patients	Clinical Trial Phase I (NCT04374877)	(163)
Norcantharidin-carrying MSC-based exosome	Effectively delivers anti-HCC tumor drugs to the liver and persists in damaged liver lesion	Pre-Clinical	(164)
	Inhibits tumorigenic capacity of HCC cells through possible downregulation of miR expression		
	Repairs damaged liver through possible upregulation of hepatocyte proliferative proteins due to exosomes		
MSC-based micro engineered HCC organoid	Serves as a prediction tool of anti-tumor drug and immunotherapy efficacy towards HCC patients that mimics the immunosuppressive TME	Pre-Clinical	(165)
QNZ (EVP4593)	Blockade of TNF-α and NF-κB HCC-induced expression reduces the extent of tumorigenicity in HCC rat model	Pre-Clinical	(166)
Galunisertib (LY2157299) + Nivolumab	Safe, well-tolerable, and effective mono-therapeutic approach that targets TGF-β in HCC	Clinical Trial Phase I/II (NCT02423343)	(167)
Plerixafor (AMD3100) + Sorafenib	Delays HCC progression and prevents infiltration of immunosuppressive cells under persistent hypoxic TME	Pre-Clinical	(168)
Plerixafor (AMD3100) + Sorafenib + anti-PD-1 treatment	Mediates anti-tumor immune response through activation and infiltration of CD8^+^ T-cells	Pre-Clinical	(168)
Plerixafor (AMD3100) + TACE	Substantially impedes HCC development and elevates apoptotic events	Pre-Clinical	(169)
	Modulates expression of CD34, HIF-1A, and VEGF that correlates with HCC progression and formation of microvesicle		
BPRCX807	Inhibits CXCR4-mediated metastasis and EMT phenotype under hypoxic condition	Pre-Clinical	(170)
	Sensitizes HCC cells to sorafenib treatment		
	Decreases the immunosuppressive extent of HCC TME through elevated infiltration of cytotoxic T-cells and reduction of TAM infiltrations		
LFC131	Sensitizes HCC cells to sorafenib treatment	Pre-Clinical	(171)
PFH@LSLP (PFH-cored liposome nanoparticles conjugated with LFC131 and PLX3397)	Enhances anti-tumor efficacy through increased infiltration of CD8^+^ T-cells and DC cells	Pre-Clinical	(171)
	Shifts immunosuppressive TME towards tumor suppressive environment		
CXCR4-targeted p53 mRNA nanoparticles + anti-PD-1 treatment	Reprograms immunosuppressive TME by enhancing potent anti-tumor immune responses by reducing the expression of immunosuppressive cytokines	Pre-Clinical	(172)
CXCR4-targeted lipid coated PLGA nanoparticles (AMD3100-based) + Sorafenib	Enhances cytotoxicity towards HCC cells leading to elevation of apoptotic events	Pre-Clinical	(173)
	Polarizes immunosuppressive TME towards tumor suppressive environment		
CXCR4-targeted PLGA-PEG nanoparticles conjugated with LFC131	Co-delivers sorafenib and metapristone into HCC cells	Pre-Clinical	(174)
	Exerts anti-tumor effect towards HCC cells by reducing CXCR4 expression and elevates therapeutic efficacy of sorafenib		

An established IL-6 antagonist known as an anti-Gr-1 antibody targets immunosuppressive MDSCs, resulting in a decrease in IL-6 levels and related immunosuppressive elements, such as arginase, NO, and ROS (101). This approach leads to the depletion of MDSCs and enhances chemotherapeutic efficacy and response. A humanized IL-8 antagonist known as BMS-986253 is currently in a phase II clinical trial (NCT04050462) in combination with nivolumab, an anti-PD-1 antibody (160). This combination therapy approach aims to improve the objective response rate of patients with advanced HCC compared with that of patients undergoing nivolumab monotherapy. Similarly, combination therapy involving BMS-986253, nivolumab, and BMS-813160 is currently in a phase II clinical trial (NCT04123379) (161). BMS-813160 is a dual CCR2/CCR5 antagonist in which these targets are known to be immunosuppressive. A substantial positive immune response and long-term survival benefit for HCC patients are expected from this clinical trial. BMS-986253 or HuMAX-IL8 alone has been tested in a phase I clinical trial (NCT02536469), with the expectation that blockade of IL-8 can degrade mesenchymal phenotypes in solid tumor cells and diminish treatment resistance (162). However, this clinical trial does not focus on HCC specifically but rather on all forms of solid tumors. Moreover, CHS-388, formerly known as SRF-388, an antagonist of the immunosuppressive agent IL-27, is being tested in a phase I clinical trial (NCT04374877) focused on developing a safe, well-tolerated, and effective monotherapeutic approach for targeting IL-27 in HCC patients (163).

MSC-based therapeutic approaches have also been documented in preclinical trials. One study investigated the delivery of norcantharidin, a potential anticancer drug in HCC patients, which is mediated by MSC-derived exosomes (164). This study demonstrated the effective delivery of anti-HCC tumor drugs to the liver, where they persist in damaged liver lesions and inhibit the tumorigenic capabilities of HCC cells through possible downregulation of miR expression (164). Moreover, this delivery system repairs the damaged liver through possible upregulation of hepatocyte proliferative proteins due to exosomes (164). In another study, norcantharidin was found to impede IL-6-induced EMT, leading to suppression of the JAK/STAT/TWIST signaling pathway (175). Interestingly, a research group successfully devised a microengineered organoid-on-a-chip through a coculture system of MSCs and peripheral blood mononuclear cells (PBMCs) in combination with CAFs and patient-derived organoids (PDOs) that can mimic the immunosuppressive TME (165). Such an innovation provides a platform for researchers to predict the outcomes of patients treated with immunotherapeutic modalities for HCC.

QNZ (EVP4593) is a relatively novel antagonist that inhibits HCC-induced TNF-α and NF-κB expression, leading to increased survival, reduced liver nodules, and an improved hepatocyte structure in the HCC rat model (166). Moreover, galunisertib (LY2157299) is undergoing a phase I/II clinical trial (NCT02423343) in conjunction with nivolumab (167). This clinical trial investigated the clinical efficacy and safety of the combination of galunisertib and nivolumab in patients with recurrent or refractory HCC. Galunisertib is an antagonist of the immunosuppressive agent TGF-β, and preliminary results have shown that galunisertib induces a vigorous antitumor immune response and mediates the breach of T cells toward the midpoint of tumors (176).

Plerixafor (AMD3100) is a potential CXCR4 antagonist that continues to be studied. AMD3100 in combination with sorafenib was found to delay the progression of HCC and impede the infiltration of immunosuppressive cells under persistent hypoxic conditions (168). Moreover, the same study incorporated AMD3100, sorafenib, and anti-PD-1 treatment, which mediated the antitumor immune response through the activation and infiltration of CD8^+^ T cells (168). TACE is still used for treating HCC patients, and a research group investigated the effects of combination therapy of AMD3100 with TACE in an HCC rat model. The results indicated a substantial hindrance to HCC development and an increase in apoptotic events, in which such a therapeutic approach can modulate the expression of CD34, HIF-1α, and VEGF, which is correlated with HCC progression and the formation of microvesicles (169). Moreover, another potent CXCR4 inhibitor known as BPRCX807 was found to inhibit CXCR4-mediated metastasis and the EMT phenotype under hypoxic conditions, sensitize HCC cells to sorafenib treatment, and decrease the extent of immunosuppression in the HCC TME through increased infiltration of cytotoxic T cells and reduced infiltration of TAMs (170). Similarly, LFC131, a CXCR4 peptide-based inhibitor, was also found to sensitize HCC cells to sorafenib treatment (171).

The utilization of a nanoparticle-based treatment approach was found to facilitate the targeting of a specific protein target, namely CXCR4. PFH@LSLP, which is a PFH-cored liposome nanoparticle conjugated with LFC131 and PLX3397, was shown to enhance antitumor efficacy through increased infiltration of CD8^+^ T cells and DCs while also shifting the immunosuppressive TME toward a tumor-suppressing environment (171). Similar results were also exhibited in a study that focused on CXCR4-targeted p53 mRNA nanoparticles in combination with anti-PD-1 treatment (172). Aside from the modified TME state from being immunosuppressive, this therapeutic approach elevates the expression of MHC-I and reduces the expression of immunosuppressive cytokines (172). AMD3100-based lipid-coated Poly (lactic-co-glycolic acid) (PLGA) nanoparticles in combination with sorafenib, which was developed by a research group, has been shown to enhance the cytotoxicity toward HCC, leading to elevated apoptotic events and polarizing the immunosuppressive TME toward a tumor-suppressive environment (173). In addition, CXCR4-targeted PLGA-PEG nanoparticles conjugated with LFC131 allow the codelivery of sorafenib and metapristone into HCC cells, which leads to antitumor effects through a decrease in CXCR4 expression and an increase in the efficacy of sorafenib treatment (174).

## 10 Conclusion

Liver CSCs have been shown to contribute significantly to therapeutic resistance and tumor relapse in HCC patients, suggesting that prevention strategies and the development of innovative treatment approaches are important. Additionally, therapeutic resistance becomes even more complex with the contribution of secretory factors derived from liver CSCs, which leads to the establishment of an immunosuppressive TME that debilitates effective antitumor immune responses. As described in this review, CSCs may participate in the secretion of immunosuppressive factors, such as cytokines, growth factors, and exosomes, which collectively make up anatomically distinct CSC niches within the TME in HCC. Although there is a lack of evidence and research on the secretion of immunosuppressive factors by CSCs, these conspicuous cells can attract and participate in crosstalk with diverse cellular components within the TME that ultimately secrete various factors. To address the immunosuppressive state of the TME, therapeutic approaches targeting these secretory factors are crucial. An in-depth analysis of these approaches is needed to provide a better understanding of how to effectively reduce the immunosuppressive state of the TME in patients with HCC while still providing beneficial, safe, and well-tolerated ICI treatment options.

## Author contributions

MK: Writing – review & editing, Writing – original draft. TK-W: Writing – review & editing, Supervision, Funding acquisition.

## Glossary

**Table:**

ABC	ATP-binding cassette
ActD	actinomycin D
AFP	alpha-fetoprotein
BCLC	Barcelona clinical liver cancer
CAFs	cancer-associated fibroblasts
CARs	chimeric antigen receptors
CK19	cytokeratin 19
COX	cyclooxygenase
CPO	chromenopyrimidinone
CSCs	cancer stem cells
CXCL	C-X-C motif chemokine ligand
CXCR	C-X-C motif chemokine receptor
DC	dendritic cell
ECM	extracellular matrix
EMT	epithelial-mesenchymal transition
EpCAM	epithelial cellular adhesion molecule
EVs	extracellular vesicles
FACS	fluorescence-activated cell sorting
FGF	fibroblast growth factor
FGFR	fibroblast growth factor receptor
FLT3	FMS-like tyrosine kinase-3
GAL	galectin
GPC3	glypican-3
HBV	hepatitis B virus
HCC	hepatocellular carcinoma
HCV	hepatitis C virus
ICIs	immune checkpoint inhibitors
IDO	indoleamine 2,3-dioxygenase
IFN	interferon
IGF	insulin-like growth factor
IL	interleukin
lncRNAs	long non-coding RNAs
MCP	monocyte chemoattractant protein
MDR	multidrug resistance
MDSCs	myeloid-derived suppressor cells
miRNAs	microRNAs
MMP	matrix metalloproteinases
MSCs	mesenchymal stem cells
NAFLD	non-alcoholic fatty liver disease
NASH	non-alcoholic steatohepatitis
NK	natural killer
NO	nitric oxide
PBMCs	peripheral blood mononuclear cells
PDGFR	platelet-derived growth factor receptor
PDO	patient-derived organoid
PGE2	prostaglandin E2
PLGA	Poly (lactic-co-glycolic acid)
ROS	reactive oxygen species
SHH	sonic hedgehog
STC	stanniocalcin
TACE	transarterial chemoembolization
TAMs	tumor-associated macrophages
TGF	transforming growth factor
TICs	tumor-initiating cells
TKIs	tyrosine kinase inhibitors
TME	tumor microenvironment
TNF	tumor necrosis factor
Tregs	regulatory T-cells
VEGF	vascular endothelial growth factor
VEGFR	vascular endothelial growth factor receptor
